# Data of transcriptional effects of the merbarone-mediated inhibition of TOP2

**DOI:** 10.1016/j.dib.2022.108499

**Published:** 2022-08-01

**Authors:** Fernando M. Delgado-Chaves, Pedro Manuel Martínez-García, Andrés Herrero-Ruiz, Francisco Gómez-Vela, Federico Divina, Silvia Jimeno-González, Felipe Cortés-Ledesma

**Affiliations:** a Division of Computer Science, Pablo de Olavide University, Seville ES-41013, Spain; b Andalusian Molecular Biology and Regenerative Medicine Center (CABIMER), University of Seville - CSIC - Pablo de Olavide University, Seville ES-41092 Spain; c Topology and DNA breaks Group, Spanish National Cancer Research Center (CNIO), Madrid ES-28029, Spain

**Keywords:** Topoisomerase inhibition, Differential gene expression, DNA supercoiling, Merbarone, RNA-Seq

## Abstract

Type II DNA topoisomerases relax topological stress by transiently gating DNA passage in a controlled cut-and-reseal mechanism that affects both DNA strands. Therefore, they are essential to overcome topological problems associated with DNA metabolism. Their aberrant activity results in the generation of DNA double-strand breaks, which can seriously compromise cell survival and genome integrity. Here, we profile the transcriptome of human-telomerase-immortalized retinal pigment epithelial 1 (RPE-1) cells when treated with merbarone, a drug that catalytically inhibits type II DNA topoisomerases. We performed RNA-Seq after 4 and 8 h of merbarone treatment and compared transcriptional profiles versus untreated samples. We report raw sequencing data together with lists of gene counts and differentially expressed genes.

## Specifications Table

**Table:** 

Subject	Molecular biology
Specific subject area	NGS, Transcriptomics
Type of data	Table
How data were acquired	RNA-Seq data acquired by Illumina NextSeq 500 (1 × 75 bp single-read sequencing)
Data format	Raw and processed data
Parameters for data collection	Total RNA was extracted and sequenced from human-telomerase-immortalized retinal pigment epithelial 1 (RPE-1) cells, treated with merbarone for 4 and 8 hours.
Description of data collection	Serum-starved human-telomerase-immortalized retinal pigment epithelial 1 (RPE-1) cells grown on 60mm plates were treated as required and total RNA was isolated with the RNeasy kit (QIAGEN, 74106), following instructions from the manufacturer. Then, total RNA (150ng) cDNA libraries were prepared using TruSeq Stranded mRNA (lllumina). Library size distribution was analyzed with Bioanalyzer DNA high-sensitive chip and Qubit. 1.4pM of each library was sequenced in NextSeq 500 HIGH-Output.
Data source location	Andalusian Molecular Biology and Regenerative Medicine Centre, Seville, Spain.
Data accessibility	RNA-Seq data (raw FASTQ, gene count tables and bigWig files) generated in this study are available under GEO accession number GSE198093. Lists of differentially expressed genes reported in this manuscript are available in the Supplementary Material.
Related research article	Andrés Herrero-Ruiz, Pedro Manuel Martínez-García, José Terrón-Bautista, Gonzalo Millán-Zambrano, Jenna Ariel Lieberman, Silvia Jimeno-González, Felipe Cortés-Ledesma Topoisomerase IIα represses transcription by enforcing promoter-proximal pausing Journal DOI: doi.org/10.1016/j.celrep.2021.108977

## Value of the Data

• Type II topoisomerases are crucial enzymes involved in the regulation of DNA supercoiling. Here we report RNA-Seq data that explain gene expression dynamics when inhibiting topoisomerase II at different time points.
• The datasets and analyses provided here can be useful for researchers focusing on transcriptional regulation, DNA damage, DNA repair and the implications of topoisomerase impairment in disease.
• The reported gene counts and differentially expressed genes tables can be used in different analyses such as clustering, bi-clustering, forecasting methods or inference of gene interaction networks.

## 1 Data Description

### 1.1 RNA-Seq samples

To study potential roles of human topoisomerase II (TOP2) in regulating transcription, we generated RNA-Seq profiles of RPE-1 cells treated with merbarone, a drug that catalytically inhibits TOP2, at different time points. In a previous work, we profiled RNA-Seq of untreated samples and upon 30 min and 2 h of merbarone treatment [1]. Here we provide transcriptional profiles at two later time points, namely 4 and 8 h of merbarone treatment. Table 1 shows a summary of libraries statistics and mapping results. In Herrero-Ruiz et al. [1], we found that few genes were affected upon 30 min of merbarone treatment. Therefore, untreated samples and samples treated during 2, 4 and 8 h were used for subsequent analysis.

**Table 1:** Summary of sequencing and mapping statistics.

Sample name	Number of raw reads	Number of mapped reads	Mapping rate
Merbarone 4h repl.1	19,897,314	12,042,000	60.52%
Merbarone 4h repl.2	19,776,837	11,866,705	60%
Merbarone 8h repl.1	18,776,616	11,214,683	59.73%
Merbarone 8h repl.2	15,490,983	9,273,789	59.87%

### 1.2 Data Analysis

**Fig. 1:**
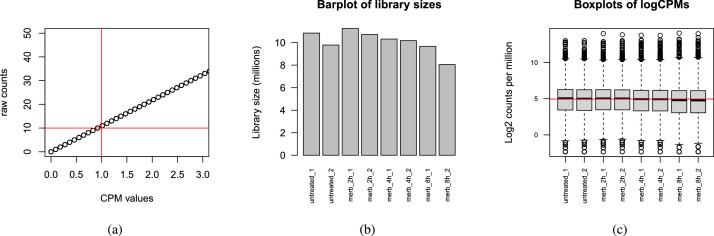
(a) Correspondence between raw counts and CPM values. (b) Comparison of library sizes, in million of raw counts. (c) Comparison of library sizes in log2-CPM, which corrects for differences in library size.

We selected and removed lowly expressed genes as those not overcoming a minimum counts per million (CPM) threshold in at least 2 samples. Since we had 2 biological replicates, the smallest sample size for each group in this experiment was 2. In this dataset, we chose CPM threshold of 1, as shown in Fig. 1a, which retained as a result around 55% of the initial genes.

The exploration of library sizes revealed a non-Gaussian data distribution, as shown in Fig. 1b. In order to correct for the different library sizes, we obtained log2-CPM data (Fig. 1c), which resulted in analogous distributions for each sample. Hence, all samples proceeded to the analysis as they were considered similar compared to each other.

**Fig. 2:**
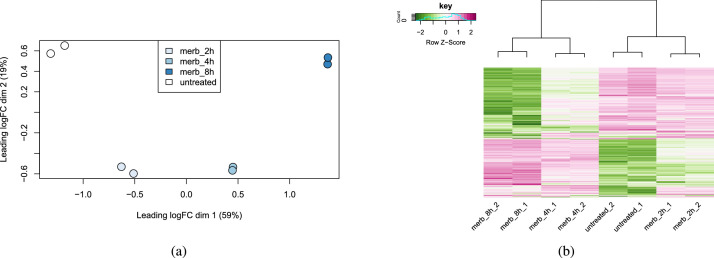
(a) MDS plot representing major differences between samples, colored according to time points. (b) Heatmap showing hierarchical clustering between samples for the top 500 most variable genes.

Multidimensional scaling (MDS) detected major differences between samples from different groups, while samples from the same group (replicates) cluster together in the plot (Fig. 2a). This is indicative of the correct performance of the experiment. Consistently with MDS, hierarchical clustering revealed greatest similarities between biological replicates (Fig. 2b). Moreover, untreated samples exhibited a similar expression pattern to samples treated with merbarone for 2h, for the top 500 most variable genes, while samples a higher time points showed greater similarities between each other. These similarities could be indicative of a progression in gene expression profile over time.

We performed differential expression analyses between untreated samples and samples at 2 h, 4 h and 8 h, respectively yielding 54, 241 and 702 DEGs. A summary of DEGs is detailed in Table 2. Analysis of DEGs revealed a cumulative effect of merbarone-mediated TOP2 inhibition over time, with an initial predominance of genetic down-regulation. In Supplementary Material, we provide the complete list of DEGs at each time point vs. untreated samples.

**Table 2:** Summary of DEGs for the three performed comparisons.

	untreated - merb_2h	untreated - merb_4h	untreated - merb_8h
**Up**	3	50	316
**Down**	51	191	386
**Total**	54	241	702

## 2 Experimental Design, Materials and Methods

### 2.1 Cell cultures

hTERT RPE-1 cell (ATCC) were cultured in Dubelccos Modified Eagles Medium (DMEM) F-12 (Sigma) supplemented with penicillin, streptomycin 50 units ml-1 each and 10% Fetal Bovine Serum (FBS) (Sigma) at 37æC in 5% CO_2_ atmosphere. For experiments, RPE-1 cells were washed with PBS and G0/G1-arrested by serum starvation during 48h using 0.1% FBS-containing DMEM media supplemented with antibiotics. Then, cells were treated with 200μM merbarone (Sigma) or DMSO for the indicated times. The presence of mycoplasma was frequently checked with MycoAlert PLUS Mycoplasma Detection Kit (Lonza).

### 2.2 RNA Isolation and Library Preparation

48h Serum-starved RPE-1 cells grown on 60 mm plates were treated as required and total RNA was directly isolated with the RNeasy kit (QIAGEN), following instructions from the manufacturer.

The cDNA libraries for RNA-Seq were prepared using TruSeq Stranded mRNA (lllumina) following manufacturer’s protocol. Library size distribution was analyzed with Bioanalyzer DNA high-sensitive chip and quantified by Qubit. Finally, 1.4 pM of each library was sequenced in NextSeq 500 HIGH-Output.

### 2.3 Sequencing Data Analysis

Sequence reads were demultiplexed, quality filtered with *FASTQC* [2] and mapped to the human genome (hg19) using Bowtie 1.2 [3]. We used option ’-m 1’ to retain only those reads that map only once to the genome. Count data resulting from mapping at the gene level is provided in the Supplementary Material.

### 2.4 Count Data Pre-processing

Data preprocessing was performed in order to reduce noise and leverage subsequent analyses. In this case, data preprocessing included removal of lowly expressed genes and log2-normalization.

First, we removed lowly expressed genes, i.e. those genes with very low counts across all samples. Such genes represent a computational burden for downstream analyses and introduce a certain bias to the multiple testing for the later estimation of differentially expressed genes. We used the *cpm* function from the *edgeR* library to obtain CPM values [4]. Conventionally, an appropriate CPM threshold corresponds to a sequencing depth of 10, which we used.

### 2.5 Count data exploration

We roughly explored cross-sample differences via unsupervised analyses such as MDS and hierarchical clustering with heatmaps. MDS plots are a visualization of a principal components analysis, an unsupervised method that determines the main sources of variation in data. We generated MDS plots using the *plotMDS* function from the *limma* package [5].

Additionally, we used hierarchical clustering to assess sample similarity by estimating an euclidean distance matrix from the log-CPM data for the 500 most variable genes. In order to represent the hierarchical clustering, we generated heatmaps using the *heatmap.2* function included in the *gplots* package [6].

Finally, since libraries were different in size, we corrected for composition bias between libraries through TMM normalization. We estimated normalization factors for each library and computed the effective library size using the *calcNormFactors* function from the *edgeR* package [4].

### 2.6 Differential Expression Analysis

Normalized data proceeded to the identification of differentially expressed genes (DEGs). In this particular case, we obtained differentially expressed genes between untreated samples and merbarone-treated samples at 2h, 4h and 8h, respectively. Such comparisons allow quantifying the effect of TOP2 inhibition over time and the main genes involved in the response to merbarone. We performed differential expression analysis using the *Limma-Voom* workbench [5], [7]. We selected log2 fold change (log2FC) threshold of 1, which corresponds to double or half changes in the gene expression level, and a P value cutoff of 0.05, as adjusted by the Benjamini-Hochberg method [8].

## Ethics Statement

Neither human nor animal participants, nor the gathering of data from social media sites, were used in this study.

## CRediT authorship contribution statement

**Fernando M. Delgado-Chaves:** Data curation, Visualization, Investigation, Writing – review & editing. **Pedro Manuel Martínez-García:** Data curation, Methodology, Writing – review & editing. **Andrés Herrero-Ruiz:** Methodology, Investigation, Writing – review & editing. **Francisco Gómez-Vela:** Writing – review & editing. **Federico Divina:** Writing – review & editing. **Silvia Jimeno-González:** Conceptualization, Investigation. **Felipe Cortés-Ledesma:** Supervision, Conceptualization.

## Declaration of Competing Interest

The authors declare that they have no known competing financial interests or personal relationships which have, or could be perceived to have, influenced the work reported in this article.

## Data Availability

Gene Expression Omnibus (Original Data) GSE198093. Gene Expression Omnibus (Reference Data) GSE141799.
